# Community acquired *Pseudomonas* meningoencephalitis in a middle-aged diabetic woman: A case report

**DOI:** 10.1016/j.idcr.2025.e02206

**Published:** 2025-03-31

**Authors:** Zahra Sheidae Mehne, Ali Akbar Heydari, Kiana Ketabi, Ali Samimi, Elham Honarjou

**Affiliations:** a Department of Infectious Diseases and Tropical Medicine, Faculty of Medicine, Mashhad University of Medical Sciences, Mashhad, Iran; b Student Research Committee, Mashhad University of Medical Sciences, Mashhad, Iran

**Keywords:** Pseudomonas, Meningoencephalitis, Headache, Irritability

## Abstract

*Pseudomonas* species are environmental Gram-negative bacteria recognized for their potential to cause opportunistic infections, particularly among immunocompromised patients and those with a history of surgical procedures. Community-acquired meningoencephalitis resulting from *Pseudomonas* infection is rare but poses a significant risk of mortality. This case describes an unusual instance of primary *Pseudomonas* meningoencephalitis in a patient with diabetes mellitus, highlighting the necessity of early recognition and timely management.

## Introduction

Pseudomonas species are ubiquitous Gram-negative bacteria known for their role in opportunistic infections [1]. They are primarily associated with respiratory, urinary, and skin infections, particularly in individuals with compromised immune systems and hospitalized patients [2]. Primary infections of the central nervous system (CNS) caused by *Pseudomonas* species are rare and often occur secondary to a history of head trauma or surgical interventions [3], [4]. In this report, we present a case of primary *Pseudomonas* meningoencephalitis in a 45-year-old woman, emphasizing the importance of early diagnosis and treatment.

## Case presentation

A 45-year-old female was admitted to the emergency department with three-week history of severe and progressive fever, chills, and headache. She also reported worsening rhinorrhea, characterized by clear fluid, and episodes of vomiting. One day prior to admission, she exhibited disorientation, extreme aggression, and delusional behavior. Notably, she had not experienced rhinorrhea since hospital admission. Her relatives denied any history of cough, dyspnea, and recent head trauma.

Past medical history included poorly controlled diabetes mellitus, hypertension, and a significant accident four years earlier, which resulted in multiple bone fractures, requiring surgical intervention and the implantation of platinum prostheses in the lumbar spine and hip. The most recent surgery occurred one year ago. Her current medication history included metformin and losartan taken daily.

## Physical examination

Upon admission, the patient was cooperative and provided reliable information. Her vital signs included a pulse rate of 84/min, blood pressure of 115/70 mmHg, respiratory rate of 16/min, and temperature of 37.1 °C. Her general survey was normal, and her Glasgow coma scale was 15/15. Neurological examination revealed irritability without any focal deficits. Additionally, there were signs of increased irritability and stiff neck in response to neck flexion. The patient was subsequently hospitalized in the infectious diseases unit for further evaluation.

## Diagnostic assessments

The laboratory investigations revealed a white blood cell (WBC) count of 23,000/mm^3^ with 94 % neutrophils, hemoglobin levels of 11.5 g/dl, and platelets count of 308 × 10^9^/L. Blood urea was 23 mg/dl, serum creatinine 1 mg/dl, blood glucose 310 mg/dl. The erythrocyte sedimentation rate (ESR) was 34 mm/hr, and C-reactive protein was elevated at 146.2 mg/l.

**Fig. 1:**
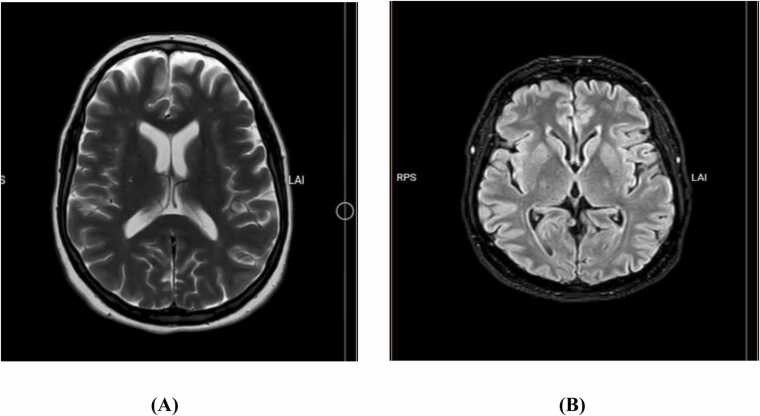
MRI in T2 (A) and FLAIR (B) sequences.

**Fig. 2:**
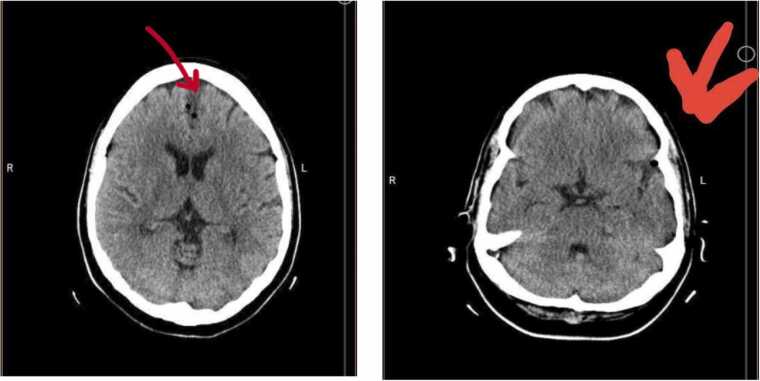
CT showing diffuse gas density in the brain parenchyma (red arrows).

Brain magnetic resonance imaging (MRI) demonstrated small foci of increased signal on T2 and fluid-attenuated inversion recovery (FLAIR) sequences in the midbrain and bilateral periventricular frontal horns, suggesting the small vessel involvement with cavum interpositum (Fig. 1). Additionally, brain computed tomography (CT) revealed diffuse gas density within the brain parenchyma, suggesting pneumocephalus (Fig. 2).

Cerebrospinal fluid (CSF) evaluation revealed semi-turbid appearance, with a leukocyte count of 5635 cells/mm³ (of which 90 % were polymorphonuclear leukocyte). Protein levels of 98 mg/dL and glucose concentration was 107 mg/dL in comparison to a blood glucose level of 310 mg/dL. Gram-staining of CSF identified Gram-negative organisms, and subsequent culture results confirmed *Pseudomonas* species sensitive to Amikacin, Cefepime, Ceftazidime, Ciprofloxacin, Meropenem, Minocyline, Piperacillin-Tazobactam, and Tobramycine, while exhibiting intrinsic resistance to Trimethoprim-Sulfamethoxazole and Ceftriaxone.

The patient promptly started on intravenous antibiotics and supportive treatment including steroids, sedative medications, seizure precautions, and proton-pump inhibitors. Blood glucose levels were managed with insulin therapy. Within 24 h of initiating treatment, the patient's symptoms gradually resolved, characterized by increased alertness and a reduction in aggressive behaviors. On the 7th day of admission, the laboratory results were as follows:

WBC count 11,920/mm^3^ with 69.3 % neutrophils, hemoglobin levels 10.50 g/dl, and platelets count 332 × 10^9^/L, blood urea 26 mg/dl, serum creatinine 0.8 mg/dl, blood glucose 214 mg/dl, and C-reactive protein 16.90 mg/l.

## Discussion

*Pseudomonas* species are considered atypical pathogen for bacterial meningitis in adults [5]. In Iran, *Pseudomonas aeruginosa* has been reported as a cause of less than 5 % of acute bacterial meningitis [6], [7]. *Pseudomonas* meningitis is a life-threatening condition with a high rate of mortality, though it has been rarely documented. Most cases are nosocomial and occurred in patients with head injuries or those who undergone neurosurgical interventions [8].

A study by Juhi et al. identified 10 cases (9.9 %) of nosocomial *Pseudomonas* meningitis, among the 121 cases of postsurgical meningitis [9]. Similarly, Huang et al. and Erdem et al. also reported *Pseudomonas aeruginosa* was responsible for 8.3–10.7 % of meningitis cases in post-neurosurgical patients [10], [11]. Additionally, a recent study by Hazim et al. reported a case of *Pseudomonas* Meningitis following spinal anesthesia [12].

*Pseudomonas* is recognized as an opportunistic pathogen, frequently associated with infections in individuals with compromised immune systems [13]. In the case presented, although she had no prior history of trauma or neurological procedures, the patient's poorly controlled diabetes likely contributed to the development of *Pseudomonas* meningoencephalitis. This aligns with the report by Kamath et al. which described the first case of spontaneous community-acquired *Pseudomonas* meningitis in a diabetic female without any prior history of head injury or neurosurgical interventions [8].

Another interesting aspect of our case is the simultaneous presentation of both meningitis and encephalitis. Meningoencephalitis is a life-threatening inflammatory process that affects both the meninges and the brain parenchyma [14]. The presence of community-acquired meningoencephalitis due to *Pseudomonas* species is uncommon and is primarily documented in case reports. For instance, A study by Williams et al. reported a case of *Pseudomonas* meningoencephalitis that mimicked a stroke in an elderly woman on immunosuppressive therapy, who presented with focal neurological deficits and ultimately succumbed to the condition [15]. Another study reported a case of meningoencephalitis caused by both *Pseudomonas* and *Acinetobacter*, which was treated with intraventricular antibiotics [16].

While meningoencephalitis due to *Pseudomonas* species is extremely rare, it emphasizes the need for attentiveness in patients with underlying conditions, including diabetic patients. It also underscores the need for glycemic control to reduce the risk of opportunistic infections. Early recognition of symptoms, prompt CSF analysis, and targeted antibiotic management are critical in the patient's recovery.

## Conclusion

This case presented a rare but potentially life-threatening occurrence of *Pseudomonas* meningoencephalitis in a patient with diabetes mellitus. It underscores the importance for healthcare professionals to remain vigilant regarding atypical pathogens in patients with underlying diseases who present neurological symptoms. Early diagnosis and prompt management are essential to improve prognosis in such cases. Additionally, maintaining good glycemic control in diabetic patients may help mitigate the risk of opportunistic infections, further emphasizing the need for comprehensive patient care.

## Ethical consideration

Informed consent was obtained from the patient, allowing for the inclusion of this information in our case report.

## Funding

None

## CRediT authorship contribution statement

**Zahra Sheidae Mehne:** Writing – original draft, Project administration. **Ali Akbar Heydari:** Investigation. **Kiana Ketabi:** Writing – review & editing, Supervision, Software. **Ali Samimi:** Formal analysis, Data curation. **Elham Honarjoue:** Supervision.

## Declaration of Generative AI and AI-assisted technologies in the writing process

In the development and composition of this document, generative AI was not utilized beyond the scope of fundamental grammar, spelling, and reference verification tools.

## Conflicts of interest

The authors have no conflicts of interest in this article.
